# Gut microbiota was modulated by moxibustion stimulation in rats with irritable bowel syndrome

**DOI:** 10.1186/s13020-018-0220-y

**Published:** 2018-12-18

**Authors:** Xiaomei Wang, Qin Qi, Yuanyuan Wang, Huangan Wu, Xiaoming Jin, Huan Yao, Duiyin Jin, Yanan Liu, Cun Wang

**Affiliations:** 1 Shanghai Research Institute of Acupuncture and Meridian, Shanghai University of Traditional Chinese Medicine, 650 South Wanping Road, Xuhui District, Shanghai, 200030 China; 2 Key Laboratory of Acupuncture and Immunological Effects, Shanghai University of Traditional Chinese Medicine, Shanghai, 200030 China; 3 Yueyang Clinical Medical College, Shanghai University of Traditional Chinese Medicine, Shanghai, 200437 China; 4 Stark Neurosciences Research Institute & Department of Anatomy and Cell Biology, Indiana University School of Medicine, Indianapolis, IN 46202 USA; 5 Department of Radiation Oncology, Indiana University School of Medicine, Indianapolis, IN 46202 USA

**Keywords:** Irritable bowel syndrome, Moxibustion, Gut microbiota, 16S rRNA

## Abstract

**Background:**

The pathogenesis of irritable bowel syndrome (IBS) is closely related to intestinal dysbacteriosis and can be controlled by moxibustion treatment. However, the mechanism underlying the therapeutic value of moxibustion in IBS treatment remains unknown.

**Methods:**

An IBS rat model was established by colorectal distention (CRD) stimulus and mustard oil clyster. Sixty-five male rats were randomly divided into six groups: normal, IBS model, moxibustion, electroacupuncture (EA), Bifid-triple Viable Capsule (BTVC) and Pinaverium Bromide (PB) groups. The moxibustion group was treated with mild moxibustion at the bilateral Tianshu (ST25) and Shangjuxu (ST37) for 10 min/day for 7 days, the EA group was given EA at ST25 and ST37 once daily for 7 days, while the BTVC group and PB groups received Bifid-triple Viable Capsule and Pinaverium Bromide solution (at the proportion of 1:0.018) respectively by gavage once daily for 7 days. After the treatment, abdominal withdrawal reflex (AWR) scores were determined based on CRD stimulus, gut microbiota profiling was conducted by 16S rRNA high-throughput sequencing.

**Results:**

Irritable bowel syndrome model rats had significantly increased AWR scores at all intensities (20, 40, 60 and 80 mmHg) compared with the normal group. Moxibustion treatment significantly reduced AWR scores compared with the IBS model group at all intensities. Across all groups the most abundant phyla were *Bacteroidetes* and *Firmicutes* followed by *Proteobacteria* and *Candidatus Saccharibacteria.* At genus level IBS model rats had a higher abundance of *Prevotella*, *Bacteroides* and *Clostridium XI* and a lower abundance of *Lactobacillus* and *Clostridium XIVa* compared with normal rats. These changes in microbiota profiles could however be reversed by moxibustion treatment. Alpha diversity was decreased in IBS model rats compared with normal rats, yet significantly increased in moxibustion- and PB-treated rats compared with IBS rats.

**Conclusion:**

Our findings suggest that moxibustion treats IBS by modulating the gut microbiota.

**Electronic supplementary material:**

The online version of this article (10.1186/s13020-018-0220-y) contains supplementary material, which is available to authorized users.

## Background

Irritable bowel syndrome (IBS) is one of the most common gastrointestinal disorders, affecting 10–20% of the population worldwide [1, 2]. IBS is characterized by chronic (continuous or intermittent) abdominal pain, bloating, changes in bowel habit and/or stool property. IBS has a multifactorial etiology that may include colonic dysmotility [3], visceral hypersensitivity [4], brain–gut interactions [5], genetic factors [6], post-infectious low-grade inflammation [7] and altered gut microbiota [8].

Along with the development of microecology theories, the role of the gut microbiota in IBS has been paid increasing attention in recent years. There are trillions of bacteria in the human gut that have co-evolved with us [9]. The predominant phyla in the human gut are *Firmicutes* and *Bacteroidetes*, followed by *Proteobacteria*, *Actinobacteria*, *Fusobacteria* and *Verrucomicrobia* [10]. The human gut is home to a rich variety of microbes. Accordingly, the human intestinal track, particularly the colon, is equipped with sophisticated regulatory mechanisms that facilitate intestinal balance despite complex interaction with the gut microbiota. However, once intestinal balance is disturbed chronic diseases including inflammatory bowel disease [11], allergic diseases [12], obesity [13], colorectal cancer [14] among others [15] may ensue. IBS is closely linked to alterations in gut microbiota composition [16], which can lead to increased permeability of the intestinal mucosal barrier and modulation of cytokine secretion, thus playing a significant role in the pathophysiology of IBS.

Patients with IBS generally have a reduced quality of life [17], underscoring the importance of addressing these symptoms. The treatment of IBS ranges from pharmaceutical to psychological intervention [18]. However, long-term use of currently prescribed therapeutics, such as 5-hydroxytryptamine receptor (5-HT_3_) antagonists, although partly effective, does have several side effects. Psychological treatment does not have any side effects but it is difficult to apply effectively long-term. Moxibustion is a traditional Chinese therapy used to improve general health and treat chronic conditions by stimulating specific points with heat generated by burning herbal preparations containing dried mugwort leaves [19]. Both temperature-related mechanisms and nontemperature-related mechanisms likely underlie the effects of moxibustion. The latter includes smoke, herbs, and far infrared effects [20]. Growing evidence supports moxibustion as a safe and effective treatment for IBS [21]. Interestingly, moxibustion has been shown to regulate intestinal microbiota [22]. However, few studies have explored the effect of moxibustion on the intestinal microbiota. We therefore used high-throughput sequencing to determine changes in intestinal microbial community structure in an IBS rat model with or without moxibustion treatment. Our results provide new leads regarding the pathogenesis and treatment of IBS.

## Materials and methods

The Minimum Standards of Reporting Checklist (Additional file 1) contains details of the experimental design, and statistics, and resources used in this study.

### Experimental animals

A total of 65 specific-pathogen free 8-day-old male Sprague–Dawley rats were provided by the Department of Laboratory Animal Science of Shanghai University of Traditional Chinese Medicine. The animals were raised under standard conditions at 25 ± 1 °C with a relative humidity of 50–70% and 12 h light/dark cycle. The rats did not separate from their mother until they were 4 weeks old. All rats were randomly divided into six groups: normal (n = 11), model (n = 11), moxibustion (n = 11), electroacupuncture (EA, n = 10), Bifid-triple Viable Capsule (BTVC, n = 11) and Pinaverium Bromide (PB, n = 11). All animal work was performed according to the protocols approved by the University Animal Care and Use Committee of Shanghai University of Traditional Chinese Medicine [IACUC protocol number: SYXK (Shanghai) 2009-0082] to reduce pain and to avoid damage. All efforts were made to minimize animal suffering. During establishing IBS model rats, operations should be slow and soft to avoid causing pain and distress. After the procedure, the animals were monitored until fully free to move and eat. For animal therapy, be gentle when catching animals, and take appropriate treatment after the animals calm down. At the end of the experiment, animals received a lethal dose of pentobarbital sodium to minimize animal suffering.

### Establishment of the IBS rat model

The IBS rat model was established by colorectal distention (CRD) through mechanical and chemical stimulus as previously described [23]. An inflatable balloon (Shanghai Dinghuang Industrial Co., Ltd. China) was slowly inserted rectally about 2 cm into the descending colon of rats. The balloon was distended with 0.5 ml of air, for 1 min and then repeated after 30 min. The same distention was performed for 14 consecutive days between the age of 8 and 21 days. After 4 weeks rest, mustard oil (0.2 ml, 4%, Shanghai Zhixin Chemical Co., Ltd. China.) was injected into the descending colon from the anus once a day for 14 days.

### Treatment groups

After successful establishment of the model, rats in the moxibustion group, EA group, BTVC and PB group received their relevant treatments. For the moxibustion group, the ignited moxa stick (0.5 cm in diameter) (Nanyang Hanyi Moxa Co., Ltd. China) was placed 2 cm above the bilateral Tianshu (ST25) and Shangjuxu (ST37) acupoints for 10 min/day for 7 days. ST25 is located bilaterally 5 mm lateral to the intersection between the upper 2/3 and the lower 1/3, in the line between the xiphoid process and the pubic symphysis upper border and ST37 is 5 mm lateral to the anterior tubercle of the tibia and 15 mm below the knee joint [24].

The EA group was given EA at the bilateral Tianshu and Shangjuxu acupoints with Han’s Acupoint Nerve Stimulator (Beijing Huawei Industrial Development Corporation. China. LH402A) for sparse–dense waves (frequency of sparse wave: 2 Hz, frequency of dense wave: 10 Hz, intensity: 4 mA) for 20 min, once daily for 7 days. The BTVC and PB groups received Bifid-triple Viable Capsule (Inner Mongolia Shuangqi Pharmaceutical Co., Ltd. China. Lot number: S19980004) and Pinaverium Bromide (Abbott Healthcare SAS. France. Lot number: H20120127), respectively by gavage, once daily for 7 days. The BTVC and PB solutions were prepared as specified for a weight ratio of 1:0.018 for an adult (70 kg) and a rat (200 g). Prepare the required dose of suspension with drinking water. The BTVC solution concentration was 2 mg/ml with a daily dose of 20 mg/kg. The PB solution concentration was 5 mg/ml with a daily dose of 50 mg/kg. The normal and model groups did not receive any treatment. Two rats were died in BTVC group during the treatment by gavage.

### Abdominal withdrawal reflex (AWR) scores

Abdominal withdrawal reflex scores were calculated to assess colon sensitivity to CRD after treatments according to Al-Chaer et al. [23]. Distention was produced by inflating a balloon inside the descending colon through the anus; the inflation balloon had four pressure grades: 20, 40, 60 and 80 mmHg. Each CRD lasted about 20 s and was repeated three times. AWR scores were produced blindly with no subjective judgment. The mean score for each rat was used for downstream analysis. The detailed grading rules on AWR scores are as follows: (0) no behavioral response to CRD; (1) occasional head movement at the onset of the stimulus; (2) mild abdominal muscle contraction but no lifting; (3) strong abdominal muscle contraction and the abdomen but not pelvic structure being lifted off the platform; (4) body arching and lifting of pelvic structures off the platform.

### Preparation of fecal and colon tissue samples

After calculating the AWR scores, rats were weighed and injected with 2% pentobarbital sodium (Sigma. USA. P3761). The colon samples (5 cm above the anus, 3 cm in length) were rapidly collected from the descending colon, 5 g fecal matter was collected and stored at − 80 °C for 16S rRNA sequencing. Then, colon samples were fixed in 10% paraformaldehyde for hematoxylin–eosin staining for histopathological observation.

### Fecal DNA extraction

Bacterial genomic DNA was extracted from all fecal samples using the QIAamp DNAMini Kit (QIAGEN, Germany) according to the manufacturer’s instructions. First, 100 mg fecal sample and 1.4 ml buffer ASL were added to a 2 ml tube. Next, 20 μl proteinase K was added to the tube and mixed well before incubation at 56 °C until the sample was fully dissolved. Next 200 μl buffer AL was added to the tube, mixed thoroughly, followed by incubation at 70 °C for 10 min. Subsequently, 200 μl ethanol (96%) was added to the mixture, which was then loaded onto the QIAamp Mini spin column and centrifuged at 8000 rpm for 1 min. The column material was washed with 500 μl buffer AW1 and centrifuged at 8000 rpm for 1 min, then with 500 μl buffer AW2 and centrifuged at 14,000 rpm for 3 min. Finally, the DNA was eluted in 100 μl of AE elution buffer. DNA integrity and fragment size range was assessed by agarose gel electrophoresis, and DNA concentrations were measured using a NanoDrop ND-2000 spectrophotometer (Thermo Fisher Scientific, USA).

### Illumina MiSeq sequencing

The V3–V4 region of the bacterial 16S rRNA gene was amplified by polymerase chain reaction (PCR) using universal bacterial primers 341F and 806R [25]. Pooled amplicons were sequenced on a 300 PE Illumina MiSeq. Demultiplexed reads were quality filtered based on sequence length and quality as previously described [26]. Operational taxonomic units (OTUs) were clustered at 97% similarity, and chimeric sequences were removed using UCHIME [27]. Finally, taxonomic assignment of representative sequences was preformed using the Ribosomal Database Project (RDP) MultiClassifier tool [28].

### Statistical analyses

AWR scores was analysed using SPSS21.0 software, and data were expressed as mean ± SD (Standard ± Deviation) for normally distributed data and as M (Q_25_–Q_75_) for non-normally distributed data. One-way analysis of variance (ANOVA) was performed for normally distributed data and a non-parametric test (Kruskal–Wallis *H* test.) was used for non-normally distributed data.

Bioinformatic analyses were performed using R 3.2.3 (http://cran.r-project.org). Differences in relative abundance between groups were assessed using the Kruskal–Wallis test. Alpha diversity was calculated using Simpson’s diversity index. Beta diversity was determined by analysis of similarities (ANOSIM) using unweighted UniFrac as distance metric. In addition, OTUs that are differentially abundant were determined using Linear discriminant analysis effect size (LefSE). Results were deemed significant if *P *< 0.05.

## Results

### Abdominal withdrawal reflex (AWR) scores

**Fig. 1:**
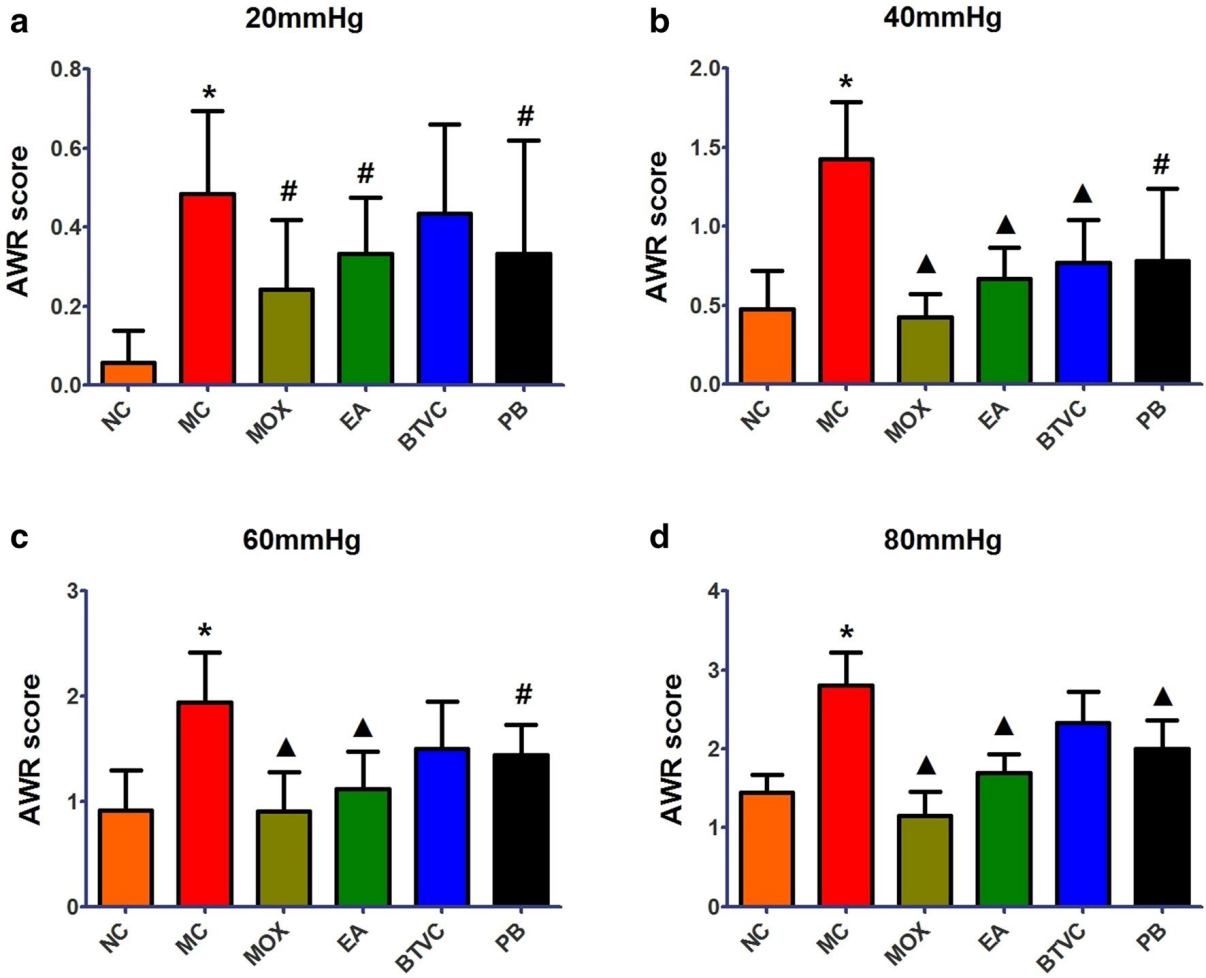
Abdominal withdrawal reflex (AWR) scores under different distention pressure (**a** 20 mmHg, **b** 40 mmHg, **c** 60 mmHg, **d** 80 mmHg) in different groups. NC: normal group; MC: IBS model group; MOX: moxibustion group; EA: electroacupuncture group; BTVC: Bifid-triple Viable Capsule group; PB: Pinaverium Bromide group. Data are presented as Median, Q_25_–Q_75_ (n = 7 per group). *P < 0.01, versus normal group; ^#^*P *< 0.05, ^▲^*P *< 0.01, versus model group

As shown in Fig. 1, AWR scores were significantly increased in IBS model rats compared with normal rats at all four CRD pressures (*P* < 0.01). AWR scores were however significantly reduced in IBS model rats following treatment with moxibustion at 20 (*P *< 0.05), 40, 60 and 80 mmHg (*P *< 0.01). AWR scores of the EA, BTVC and PB groups also were significantly lower than in IBS model group (EA group: 20 mmHg *P *< 0.05, 40, 60 and 80 mmHg, *P *< 0.01; BTVC group: 40 mmHg, *P *< 0.01; PB group: 20, 40 and 60 mmHg, *P *< 0.05, 80 mmHg, *P *< 0.01). These results suggest that moxibustion treatment could effectively decrease visceral hypersensitivity as EA, BTVC and PB.

### Histological analysis

**Fig. 2:**
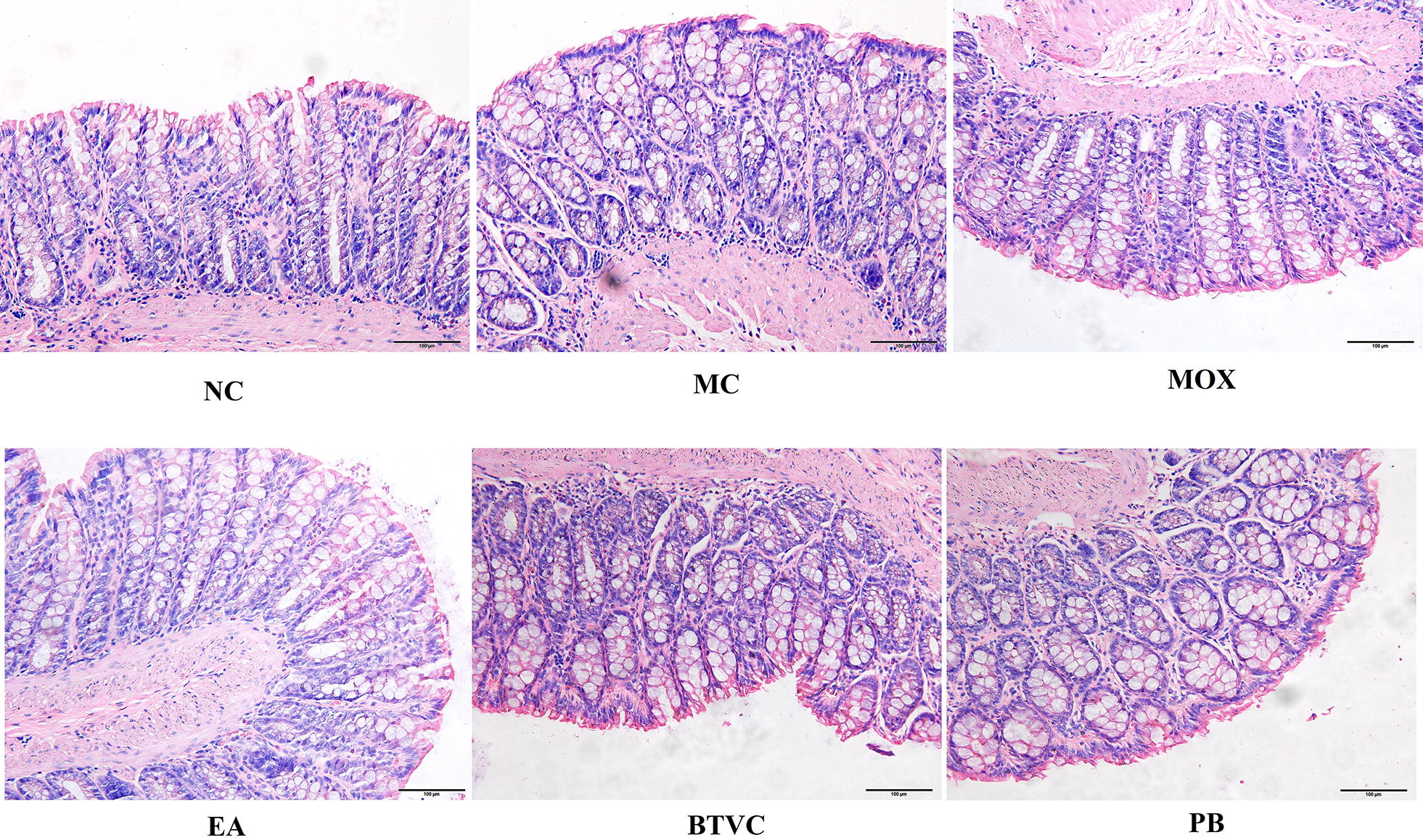
Histopathological observation of rat colonic tissue in different group. There were no significant differences in histological features between groups. NC: normal group; MC: IBS model group; MOX: moxibustion group; EA: electroacupuncture group; BTVC: Bifid-triple Viable Capsule group; PB: Pinaverium Bromide group. (magnification: ×200)

As shown in Fig. 2, there were no significant differences in histological features between groups. The colonic tissue structure was normal in all groups, and the colonic mucosa epithelium was complete and had regularly arranged glands. There was no congestion, edema, ulcers, inflammatory cell infiltration or other pathological changes in any of the groups.

### Gut microbial composition

**Fig. 3:**
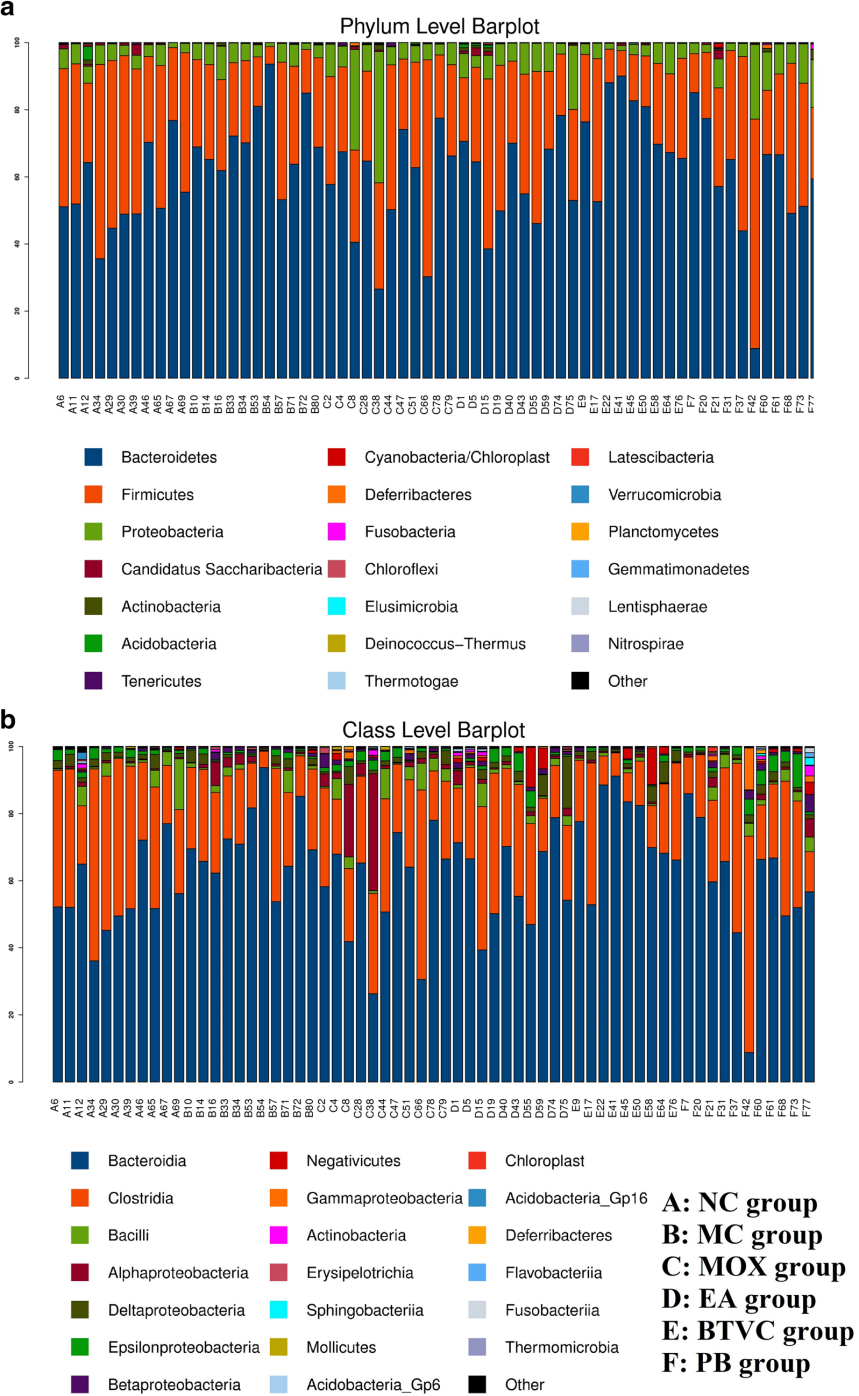
Comparison of overall community structure at phylum and class level by treatment group. **a** At phylum level, *Bacteroidetes* and *Firmicutes* were the dominant taxa across all groups, followed by *Proteobacteria* and *Candidatus Saccharibacteria*; **b** at class level, *Bacteroidia* and *Clostridia* were the dominant taxa across all groups, followed by *Bacilli* and *Alphaproteobacteria*. NC: normal group; MC: IBS model group; MOX: moxibustion group; EA: electroacupuncture group; BTVC: Bifid-triple Viable Capsule group; PB: Pinaverium Bromide group

A total of 3,759,276 high quality raw sequences were obtained using the MiSeq platform (Illumina, San Diego, CA, USA), and 2,802,729 filtered reads were retained after splicing and quality control with an average of 44,487 reads per sample (ranging from 37,081 to 54,506 reads). Reads were then clustered into OTUs at 97% similarity resulting in 1361 OTUs, which were used for further taxa diversity analysis. In terms of microbial composition, the major phyla present across all groups were *Bacteroidetes* and *Firmicutes*, followed by *Proteobacteria* and *Candidatus Saccharibacteria* (Fig. 3a). At class level, *Bacteroidia* and *Clostridia* were the dominant taxa across all groups, followed by *Bacilli* and *Alphaproteobacteria* (Fig. 3b).

### Comparison of gut microbial composition between normal and IBS model rats

**Fig. 4:**
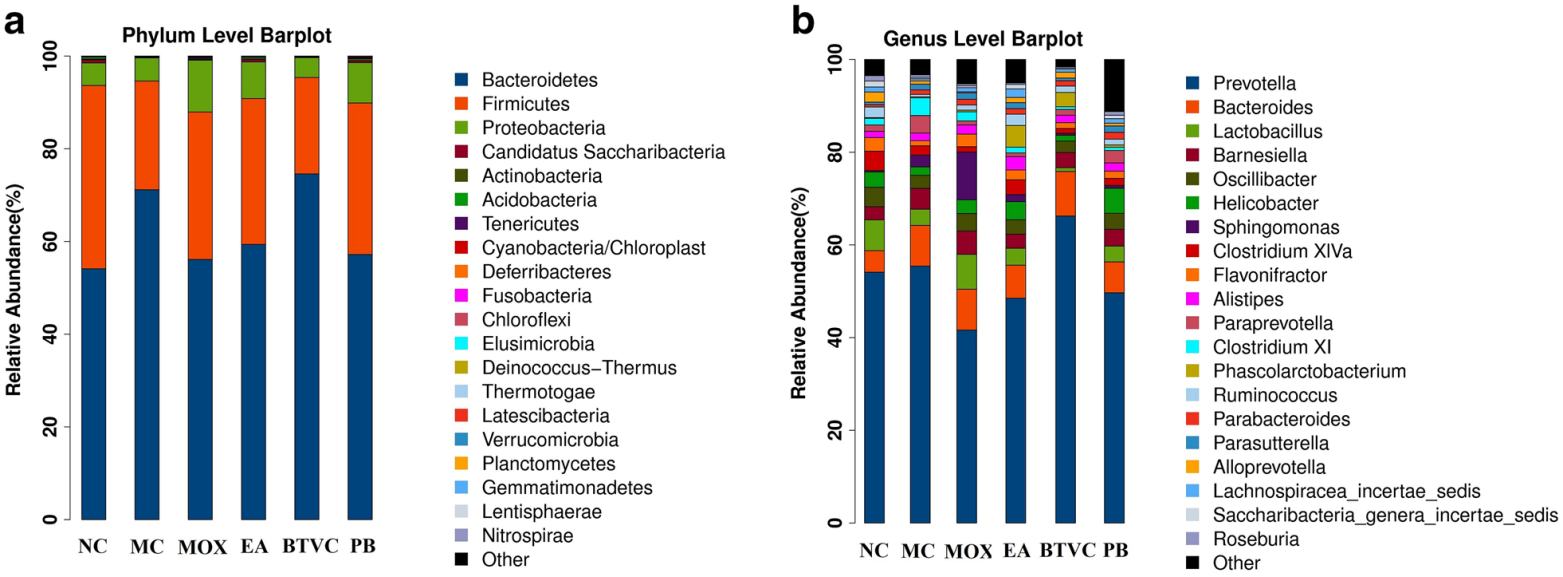
Microbiota comparison at phylum and genus level. **a** At phylum level, the relative abundance levels of *Bacteroidetes* was increased, whereas *Firmicutes* was decreased in the IBS model group compared with the normal group. These changes in microbiota profiles could be reversed by moxibustion, electroacupuncture, and Pinaverium Bromide treatment. **b** At genus level, IBS rats had decreased *Lactobacillus* and increased *Prevotella* and *Bacteroides* compared with the normal group. After treatment, *Lactobacillus* was increased and *Prevotella* was reduced in the moxibustion, electroacupuncture and Pinaverium Bromide groups. NC: normal group; MC: IBS model group; MOX: moxibustion group; EA: electroacupuncture group; BTVC: Bifid-triple Viable Capsule group; PB: Pinaverium Bromide group

At phylum level, both normal and IBS model rats had fecal samples dominated by the phyla *Bacteroidetes* and *Firmicutes*. However, the relative abundance of *Bacteroidetes* and *Firmicutes* varied significantly between groups. Compared with the normal group the model group had a higher relative abundance of *Bacteroidetes* and a lower relative abundance of *Firmicutes* (Fig. 4a). At genus level, the IBS model group had a higher relative abundance of the genera *Prevotella*, *Bacteroides*, *Barnesiella*, *Paraprevotella*, *Clostridium XI* and *Sphingomonas* compared with normal samples, and a lower relative abundance of *Lactobacillus*, *Clostridium XIVa* and *Oscillibacter* (Fig. 4b).

### Comparison of gut microbial composition between IBS model rats with and without moxibustion treatment

Treatment of IBS model rats with moxibustion led to a fecal microbial profile closer to that of normal rats, with decreased levels of *Bacteroidetes* and increased levels of *Firmicutes* following treatment (Fig. 4a). At genus level *Prevotella*, *Bacteroides* and *Clostridium XI* were decreased in moxibustion-treated IBS rats while *Lactobacillus* and *Clostridium XIVa* were increased in moxibustion-treated IBS rats (Fig. 4b). The relative abundance values were presented in Additional file 2: Table S1.

### Richness and diversity of gut bacterial communities

**Fig. 5:**
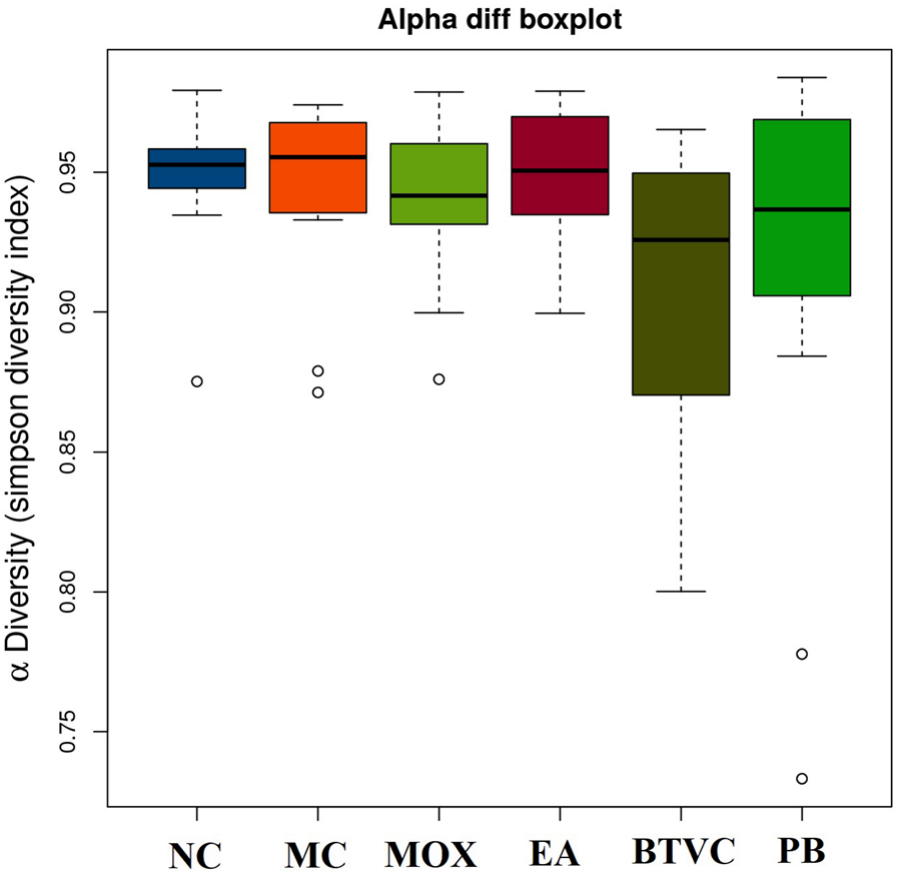
Alpha diversity of gut microbiota between groups. Alpha diversity was decreased in IBS model rats compared with normal rats, yet significantly increased in moxibustion- and Pinaverium Bromide-treated rats compared with IBS rats. NC: normal group; MC: IBS model group; MOX: moxibustion group; EA: electroacupuncture group; BTVC: Bifid-triple Viable Capsule group; PB: Pinaverium Bromide group

**Fig. 6:**
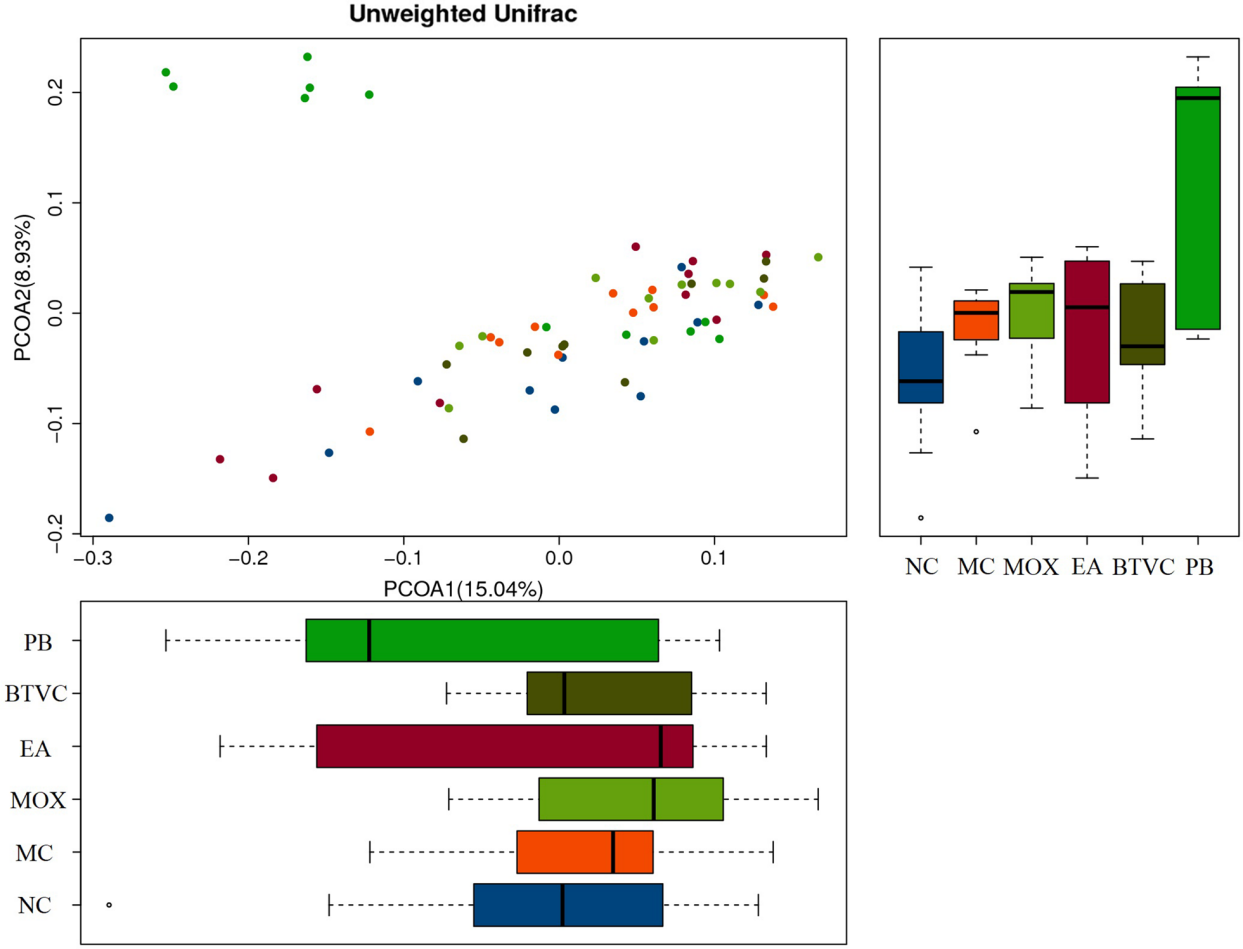
Beta diversity of gut microbiota between groups. It was showed that significant differences between normal and model groups on the second axis. NC: normal group; MC: IBS model group; MOX: moxibustion group; EA: electroacupuncture group; BTVC: Bifid-triple Viable Capsule group; PB: Pinaverium Bromide group

Alpha diversity, as measured by Simpson’s diversity index (Fig. 5), was significantly decreased in model compared with normal rats (*P* = 0.01). However, alpha diversity was increased following moxibustion treatment (*P* = 0.015). The EA, BTVC and PB groups also had higher alpha diversity than the model group, which suggests that all these treatments increase gut microbial diversity. With respect to beta diversity, principal coordinates analysis (PCoA) demonstrated significant differences between normal and model groups on the second axis (Fig. 6), suggesting that disease may be the factor influencing microbial community composition. However, the first and second principal coordinates only accounted for 15.04% and 8.93% of the total variations respectively, indicating that there are other factors affecting the IBS rats microbial community or more-refined analysis needed. The microbial community composition from EA group was more similar to PB group, and moxibustion was more similar to BTVC on the first axis.

### Fecal biomarkers of IBS and different treatments

**Fig. 7:**
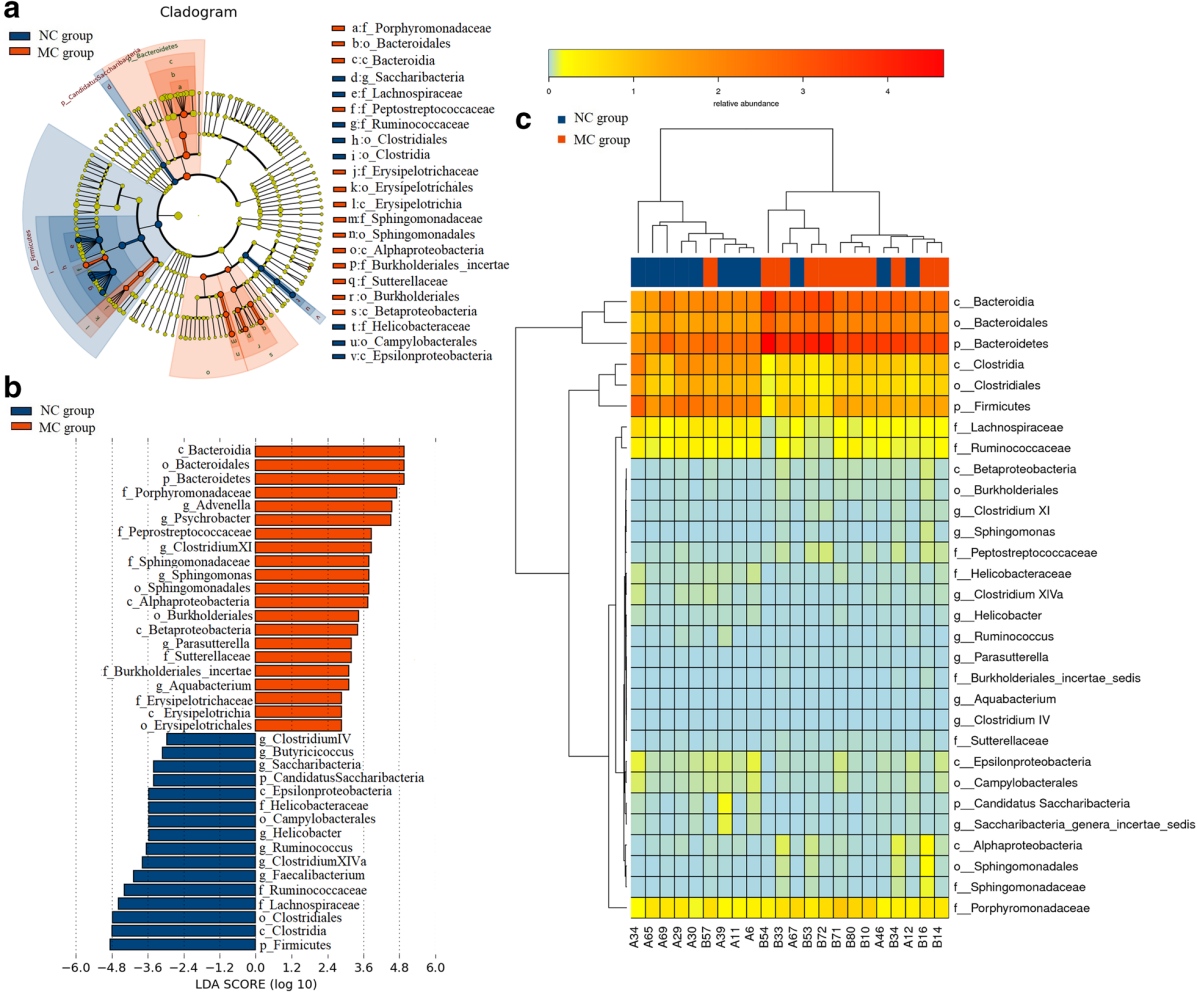
Differentially abundant OTUs between normal and IBS model rats using LEfSe analysis. **a** Taxonomic cladogram of differences in the gut microbiota between normal and IBS model groups, blue shows tax enriched in normal group, red IBS model group and yellow non-significant. The diameter of each circle is proportional to the abundance of taxon. **b** The most abundant taxa in normal group with blue histogram and IBS model group with red histogram. **c** Heatmap of OTUs found to be significantly differentially abundant between normal and IBS model rats. NC: normal group; MC: IBS model group

Linear discriminant analysis effect size (LEfSe), a biomarker discovery tool for high dimensional data, was used to determine which OTUs were differentially abundant between normal and model samples and model and different treatments samples and hence potential biomarkers of IBS and different treatments (Fig. 7a, b). A total of 37 OTUs at different taxonomic levels were differentially abundant (P < 0.05) between normal and model samples. At phylum level, the relative abundance of *Bacteroidetes* and *Firmicutes* were increased and decreased in IBS model rats, respectively. At class level *Bacterodia*, *Alphaproteobacteria*, *Betaproteobacteria* and *Erysipelotrichia* were highly enriched in IBS model rats, while *Epsilonproteobacteria* and *Clostridia* were enriched in normal rats. At order level, *Bacteroidales*, *Sphingomonadales*, *Burkholderiales* and *Erysipelotrichales* were significantly enriched in the IBS model rats. At family level *Porphyromonadaceae*, *Peptostreptococcaceae*, *Sphingomonadaceae*, *Sutterellaceae*, *Burkholderiales* and *Erysipelotrichaceae* were enriched in IBS model rats, while *Helicobacteraceae*, *Ruminococcaceae* and *Lachnospiraceae* were enriched in normal rats. Similarly, the genera *Advenella*, *Psychrobacter*, *Clostridium XI*, *Sphingomonas*, *Parasutterella* and *Aquabacterium* were significantly more abundant in IBS model rats, whereas normal rats were enriched with *Clostridium IV*, *Butyricicoccus*, *Saccharibacteria*, *Helicobacter*, *Ruminococcus*, *Clostridium XIVa*, and *Faecalibacterium.* These results are represented by heatmap analyses on a per-sample basis in Fig. 7c.

**Fig. 8:**
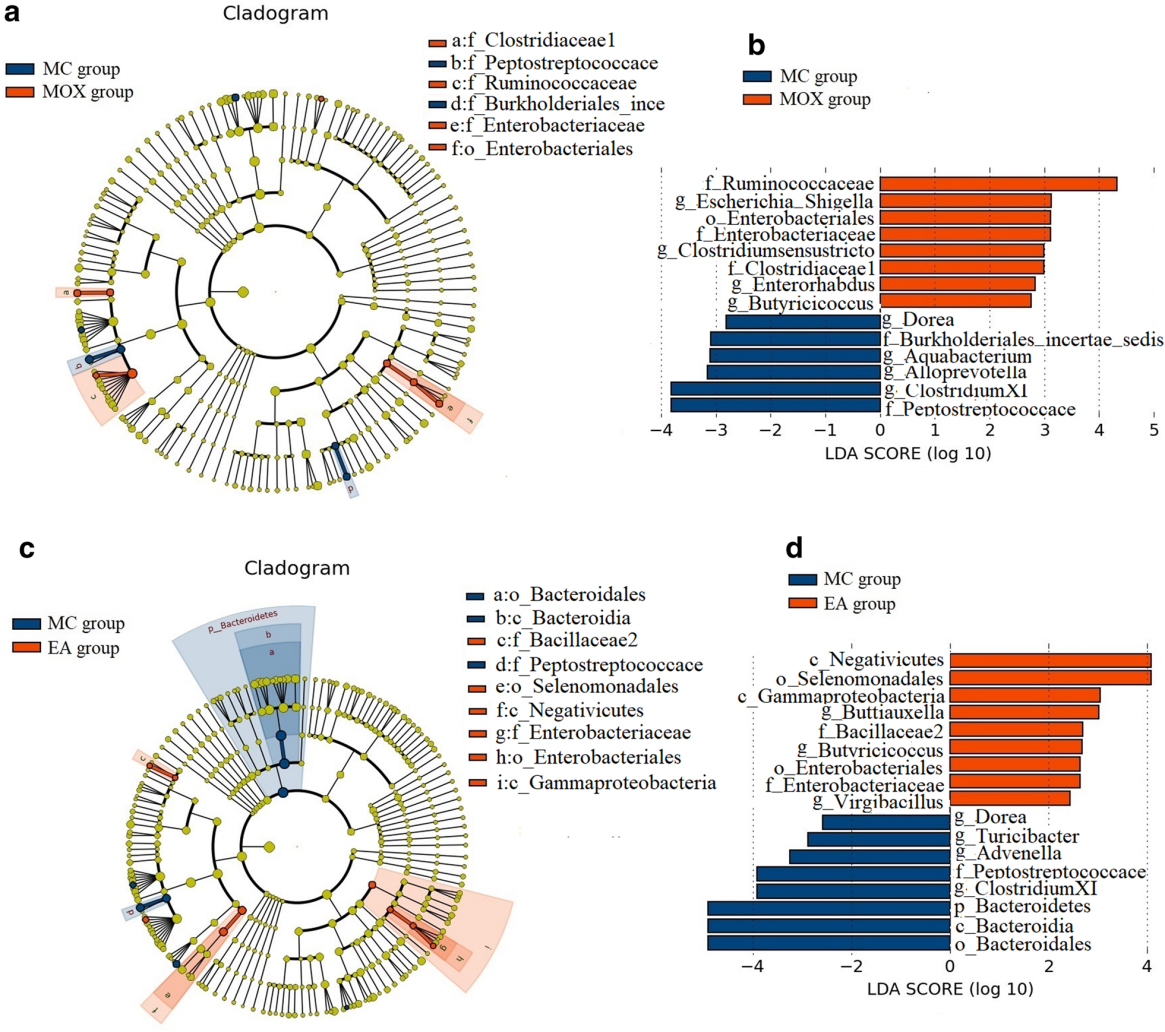
Differentially abundant OTUs between IBS and moxibustion and electroacupuncture rats using LEfSe analysis. **a** Taxonomic cladogram of differences in the gut microbiota between IBS and moxibustion groups, blue shows tax enriched in IBS group, red moxibustion group and yellow non-significant. The diameter of each circle is proportional to the abundance of taxon. **b** The most abundant taxa in IBS group with blue histogram and moxibustion group with red histogram. **c** Taxonomic cladogram of differences in the gut microbiota between IBS and electroacupuncture groups, blue shows tax enriched in IBS group, red electroacupuncture group and yellow non-significant. The diameter of each circle is proportional to the abundance of taxon. **d** The most abundant taxa in IBS group with blue histogram and electroacupuncture group with red histogram. MC: IBS model group; MOX: moxibustion group; EA: electroacupuncture group

A total of 14 OTUs at different taxonomic levels were differentially abundant (P < 0.05) between MC and MOX group. Among them, compared with MC group, the relative abundance of *Ruminococcaceae*, *Enterobacteriaceae*, *Clostridiaceae1*, *Enterobacteriales*, *Escherichia Shigella*, *Clostridiumsensustricto*, *Butyricicoccus* and *Enterorhabdus* were significant abundant in MOX group which may be the potential biomarkers of the moxibustion to treat UC (Fig. 8a, b). A total of 17 OTUs at different taxonomic levels were differentially abundant (P < 0.05) between MC and EA group. Similarly, the relative abundance of *Negativicutes*, *Selenomonadales*, *Gammaproteobacteria*, *Buttiauxella*, *Bacillaceae2*, *Butyricicoccus*, *Enterobacteriales*, *Enterobacteriaceae* and *Virgibacillus* were significant abundant in EA group which may be the potential biomarkers of the electroacupuncture to treat UC (Fig. 8c, d). Interestingly, *Butyricicoccus*, *Enterobacteriales* and *Enterobacteriaceae* were significant abundant both in MOX and EA group then MC group.

**Fig. 9:**
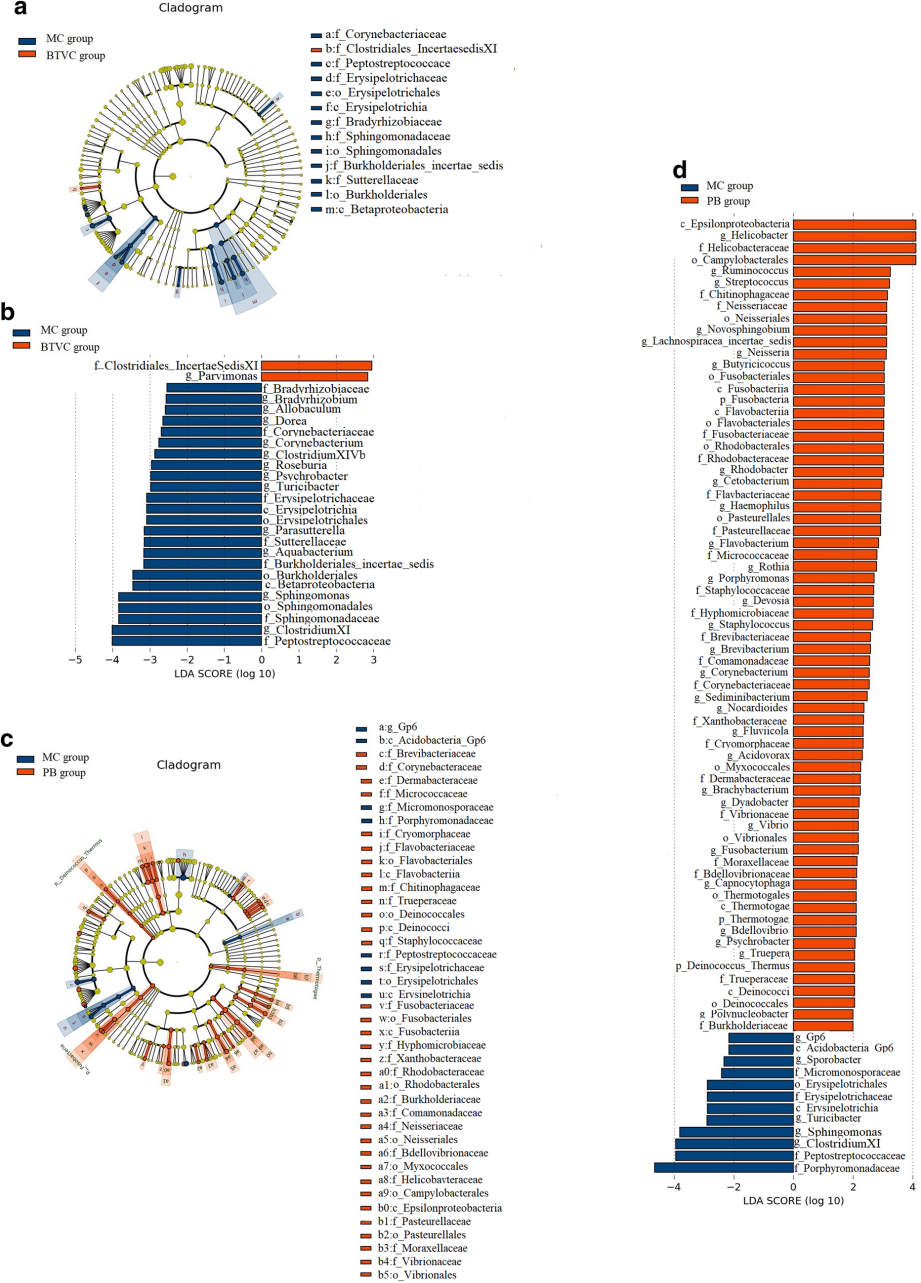
Differentially abundant OTUs between IBS and Bifid-triple Viable Capsule and Pinaverium Bromide rats using LEfSe analysis. **a** Taxonomic cladogram of differences in the gut microbiota between IBS and Bifid-triple Viable Capsule groups, blue shows tax enriched in IBS group, red Bifid-triple Viable Capsule group and yellow non-significant. The diameter of each circle is proportional to the abundance of taxon. **b** The most abundant taxa in IBS group with blue histogram and Bifid-triple Viable Capsule group with red histogram. **c** Taxonomic cladogram of differences in the gut microbiota between IBS and Pinaverium Bromide groups, blue shows tax enriched in IBS group, red Pinaverium Bromide group and yellow non-significant. The diameter of each circle is proportional to the abundance of taxon. **d** The most abundant taxa in IBS group with blue histogram and Pinaverium Bromide group with red histogram. MC: IBS model group; BTVC: Bifid-triple Viable Capsule group; PB: Pinaverium Bromide group

A total of 26 OTUs at different taxonomic levels were differentially abundant (P < 0.05) between MC and BTVC group. Among them, *Clostridiales IncertaesedisXI* and *Parvimonas* were significant abundant in BTVC group then MC group (Fig. 9a, b). A total of 81 OTUs at different taxonomic levels were differentially abundant (P < 0.05) between MC and PB group. Compared with MC group, there were 69 taxa significant abundant in PB group which may be the potential biomarkers of the Pinaverium Bromide to treat UC, such as *Ruminococcus*, *Butyricicoccus*, *Fusobacteria*, *Deinococcales*, *Thermotogae*, *Vibrionales*, *Epsilonproteobacteria* and so on (Fig. 9c, d).

## Discussion

IBS is characterized by several symptoms, including abdominal pain, that can seriously affect quality of life. Visceral hypersensitivity (enhanced intestinal perception) plays a significant role in such abdominal pain and discomfort [4]. In this study, we applied AWR scores to assess visceral hypersensitivity in rats. We found that AWR scores of IBS model rats were significantly increased compared with normal rats. Certain studies have shown that patients with IBS have a higher pain sensitivity and lower pain threshold than normal subjects [29]—our results support these findings. More importantly, AWR scores of IBS rats were significantly decreased in moxibustion, EA, BTVC and PB groups, which demonstrates that moxibustion and EA can effectively alleviated abdominal pain by increasing pain threshold and decreasing visceral hypersensitivity in IBS rats as Pinaverium Bromide and Bifid-triple Viable Capsule. Several studies have reported that electroacupuncture [30], probiotic [31] and Pinaverium Bromide [32] have therapeutic effect for IBS. Our findings indicate that moxibustion may potentially be used as an alternative treatment to Bifid-triple Viable Capsule and Pinaverium Bromide.

The intestinal microbiota profoundly affects human health through various means. Commensal bacteria promote proper functioning of the physical and biochemical barrier against pathogens as well as immune system development [33]. Intestinal bacteria and their metabolic products interact with the host gut mucosal surface thereby shaping the host immune system. Under healthy conditions the host’s response to these bacterial signals will result in immune tolerance. Normal intestinal microbiota play a critical role in promoting immune system development, sustaining normal immune function, and preventing infection by pathogens [34]. However, when dysbacteriosis occurs the balance between tolerance towards commensals and immune activation in response to pathogens may be lost, which may lead to a range of diseases.

Tianshu and Shangjuxu acupoints are ancient and classical acupoint combination for intestinal diseases such as diarrhea and abdominal pain [35]. Numerous studies suggest that dysbacteriosis is closely related to the pathophysiology of IBS [36]. Moxibustion has proven benefits in treating IBS [37]. However, ours is the first study to examine the effect of moxibustion on the gut microbiota in IBS. We analyzed changes in gut microbiota between IBS and normal rats and the effect of moxibustion therapy on the gut microbiota.

We found that the intestinal microbial composition of IBS rats differed from that of normal rats. IBS rats had significantly decreased alpha diversity and increased relative abundance of *Bacteroidetes*, which is consistent with previous reports [38]. Several studies have now reported that IBS patients and IBS model rats have significantly reduced levels of *Lactobacillus* [39, 40]. *Lactobacillus* is a major component of the commensal bacterial flora of the human intestinal tract, and is frequently used as a probiotic as it induces the production of large quantities of anti-inflammatory interleukins that improve intestinal barrier function, thus preventing the development of colitis [41]. Several studies have shown that *Lactobacillus GG*—a specific probiotic strain of *Lactobacillus* (ATCC 53103)—effectively treats IBS in humans and rats [42–44]. Indeed, in our study, *Lactobacillus* was decreased in IBS rats, as were *Clostridium XIVa* and *Oscillibacter.* Further, *Prevotella*, *Bacteroides* and *Clostridium XI* were increased in IBS model rats. Interestingly however, these IBS-related changes in gut microbiota could be normalized by moxibustion treatment, after which the relative abundance of *Lactobacillus* and *Clostridium XIVa* increased, while *Prevotella*, *Bacteroides* and *Clostridium XI* decreased. In addition, moxibustion treatment led to increased gut microbiota diversity, as did the other treatments considered in this study (EA, BTVC, and PB) to varying degrees.

We conducted LEfSe to discover distinctive features at all levels which may be the potential biomarkers of the IBS. Twenty-one features were discovered by LEfSe, and the relative abundance of *Bacteroidia*, *Bacteroidales* and *Bacteroidetes*, which exhibited the top three highest LDA score suggesting that these features may be closely related to IBS. We have also identified some potential markers that may play a therapeutic role in different treatments. It was an interesting finding that *Butyricicoccus*, *Enterobacteriales* and *Enterobacteriaceae* were significant abundant both in MOX and EA group compared to MC group. This suggests that moxibustion and electroacupuncture may have some similar therapeutic targets. Although we have found some potential biomarkers, how to regulate these markers by moxibustion and electroacupuncture still requires further research.

## Conclusions

Our findings suggest that moxibustion treats IBS by modulating the gut microbiota. We demonstrate that moxibustion could potentially be used to regulate gut microbiota imbalances and therefore to treat patients with IBS.

## Additional files

**Additional file 1.** The Minimum Standards of Reporting Checklist.

**Additional file 2: Table S1.** Microbiota comparison at phylum, class and genus levels.

## Abbreviations

IBS: irritable bowel syndrome
CRD: colorectal distention
EA: electroacupuncture
BTVC: Bifid-triple Viable Capsule
PB: Pinaverium Bromide
AWR: abdominal withdrawal reflex
5-HT_3_: 5-hydroxytryptamine receptor
PCR: polymerase chain reaction
OTUs: operational taxonomic units
RDP: Ribosomal Database Project
PCoA: principal coordinates analysis
ANOVA: one-way analysis of variance
ANOSIM: analysis of similarities
LEfSe: Linear discriminant analysis effect size
