# Supplementary material for: Gut microbiota was modulated by moxibustion stimulation in rats with irritable bowel syndrome
Source: Chin Med. 2018 Dec 18;13:63. doi: 10.1186/s13020-018-0220-y (PMC6299671; doi:10.1186/s13020-018-0220-y)
Supplement: Supplementary file 2 — Additional file 2: Table S1. Microbiota comparison at phylum, class and genus levels. [file 13020_2018_220_MOESM2_ESM.docx]

| **Phylum** | **NC** | **MC** | **MOX** | **EA** | **BTVC** | **PB** |
| --- | --- | --- | --- | --- | --- | --- |
| Acidobacteria | 0.003891 | 0.000517 | 0.000596 | 0.002284 | 0.000219 | 0.000047 |
| Actinobacteria | 0.001777 | 0.001442 | 0.002395 | 0.003147 | 0.000425 | 0.004572 |
| Bacteroidetes | 0.523270 | 0.709599 | 0.557828 | 0.567647 | 0.719894 | 0.563783 |
| Candidatus Saccharibacteria  Chlamydiae | 0.006563  0.000020 | 0.001429  0.000000 | 0.001866  0.000000 | 0.005062  0.000000 | 0.001621  0.000000 | 0.003614  0.000000 |
| Chloroflexi | 0.000488 | 0.000083 | 0.000203 | 0.000725 | 0.000101 | 0.000512 |
| Cyanobacteria/Chloroplast | 0.000532 | 0.000191 | 0.000213 | 0.000399 | 0.000098 | 0.001165 |
| Deferribacteres  Deinococcus-Thermus  Elusimicrobia  Firmicutes  Fusobacteria  Gemmatimonadetes  Latescibacteria  Lentisphaerae  Nitrospirae  Planctomycetes  Proteobacteria  Tenericutes  Thermotogae  Verrucomicrobia  Other | 0.000032  0.000015  0.000000  0.378626  0.000088  0.000020  0.000071  0.000000  0.000042  0.000061  0.041918  0.000613  0.000000  0.000047  0.041928 | 0.000034  0.000000  0.000000  0.233035  0.000034  0.000010  0.000007  0.000005  0.000002  0.000000  0.049197  0.000083  0.000000  0.000005  0.004325 | 0.001130  0.000029  0.000000  0.315400  0.000027  0.000000  0.000047  0.000002  0.000000  0.000000  0.110682  0.002258  0.000000  0.000000  0.007323 | 0.000013  0.000165  0.000008  0.300156  0.000135  0.000027  0.000000  0.000019  0.000000  0.000027  0.070945  0.000159  0.000000  0.000073  0.049009 | 0.000040  0.000003  0.000442  0.202348  0.000003  0.000000  0.000017  0.000027  0.000000  0.000000  0.040985  0.000314  0.000000  0.000000  0.033464 | 0.001008  0.000123  0.000000  0.322469  0.001959  0.000000  0.000007  0.000005  0.000000  0.000000  0.084657  0.000454  0.000221  0.000000  0.015406 |

Microbiota comparison at phylum level. NC: normal group; MC: IBS model group; MOX: moxibustion group; EA: electroacupuncture group; BTVC: Bifid-triple Viable Capsule group; PB: Pinaverium Bromide group.

| **Class** | **NC** | **MC** | **MOX** | **EA** | **BTVC** | **PB** |
| --- | --- | --- | --- | --- | --- | --- |
| Acidobacteria_Gp16 | 0.001981 | 0.000199 | 0.000027 | 0.000302 | 0.000060 | 0.000012 |
| Acidobacteria_Gp6 | 0.000848 | 0.000218 | 0.000532 | 0.001872 | 0.000195 | 0.000034 |
| Actinobacteria | 0.001714 | 0.001432 | 0.002395 | 0.003037 | 0.000437 | 0.004565 |
| Bacteroidia | 0.522735 | 0.709199 | 0.556703 | 0.566411 | 0.721085 | 0.558294 |
| Flavobacteriia | 0.000005 | 0.000015 | 0.000321 | 0.000076 | 0.000000 | 0.001866 |
| Sphingobacteriia | 0.000201 | 0.000027 | 0.000172 | 0.000418 | 0.000060 | 0.003052 |
| Thermomicrobia  Chloroplast  Deferribacteres  Bacilli  Clostridia  Erysipelotrichia  Negativicutes  Fusobacteriia  Gemmatimonadetes  Alphaproteobacteria  Betaproteobacteria  Deltaproteobacteria  Epsilonproteobacteria  Mollicutes  Other | 0.000120  0.000532  0.000032  0.031996  0.335599  0.000782  0.001226  0.000088  0.000020  0.004065  0.003172  0.017870  0.015867  0.000613  0.041928 | 0.000032  0.000191  0.000034  0.015416  0.209112  0.002241  0.001177  0.000034  0.000010  0.016362  0.008757  0.015328  0.008328  0.000083  0.004325 | 0.000086  0.000213  0.001130  0.035431  0.265801  0.002577  0.002569  0.000027  0.000000  0.063627  0.010429  0.016230  0.016583  0.002258  0.007323 | 0.000434  0.000399  0.000013  0.017783  0.251970  0.001001  0.021855  0.000135  0.000027  0.011297  0.009401  0.027823  0.019635  0.000159  0.049009 | 0.000033  0.000102  0.000108  0.005181  0.171205  0.000333  0.017682  0.000003  0.000000  0.004686  0.003668  0.022713  0.006871  0.000291  0.033893 | 0.000194  0.001165  0.001008  0.022732  0.282549  0.000772  0.006428  0.001959  0.000000  0.011611  0.012677  0.014293  0.030807  0.000454  0.015406 |

Microbiota comparison at class level. NC: normal group; MC: IBS model group; MOX: moxibustion group; EA: electroacupuncture group; BTVC: Bifid-triple Viable Capsule group; PB: Pinaverium Bromide group.

| **Genus** | **NC** | **MC** | **MOX** | **EA** | **BTVC** | **PB** |
| --- | --- | --- | --- | --- | --- | --- |
| Bacteroides | 0.024646 | 0.048057 | 0.051940 | 0.031620 | 0.050661 | 0.037373 |
| Barnesiella | 0.012560 | 0.022599 | 0.024990 | 0.014657 | 0.018617 | 0.019392 |
| Parabacteroides | 0.002555 | 0.005448 | 0.006612 | 0.004892 | 0.006406 | 0.007848 |
| Alloprevotella | 0.011182 | 0.004170 | 0.001047 | 0.005372 | 0.007422 | 0.003670 |
| Paraprevotella | 0.006318 | 0.021871 | 0.005656 | 0.004350 | 0.007695 | 0.016625 |
| Prevotella | 0.277209 | 0.319139 | 0.243357 | 0.250036 | 0.418429 | 0.313983 |
| Alistipes  Saccharibacteria  Lactobacillus  Clostridium XlVa  Lachnospiracea_incertae_sedis  Roseburia  Clostridium XI  Flavonifractor  Oscillibacter  Ruminococcus  Phascolarctobacterium  Sphingomonas  Parasutterella  Helicobacter  Other | 0.007384  0.006563  0.031751  0.021650  0.004244  0.005428  0.006320  0.002219  0.019336  0.011589  0.000733  0.001638  0.002824  0.015867  0.024157 | 0.008235  0.001429  0.015176  0.009370  0.002229  0.002856  0.021805  0.000863  0.013227  0.002292  0.000995  0.014394  0.006056  0.008328  0.020247 | 0.011067  0.001866  0.033913  0.007973  0.005045  0.002145  0.010562  0.002694  0.018723  0.006161  0.002344  0.059764  0.008063  0.016583  0.037323 | 0.012273  0.005062  0.016477  0.016240  0.008705  0.001578  0.005429  0.000995  0.015310  0.011761  0.019118  0.007554  0.005804  0.019635  0.039009 | 0.009085  0.001528  0.005046  0.006436  0.003758  0.001573  0.003131  0.001915  0.013880  0.008522  0.017523  0.002574  0.003641  0.006871  0.013893 | 0.010032  0.003614  0.018348  0.007929  0.005555  0.004420  0.003592  0.002069  0.018924  0.006639  0.002900  0.003266  0.007166  0.030807  0.065406 |

Microbiota comparison at genus level. NC: normal group; MC: IBS model group; MOX: moxibustion group; EA: electroacupuncture group; BTVC: Bifid-triple Viable Capsule group; PB: Pinaverium Bromide group.
